# Pathotypes of *Xanthomonas axonopodis* pv. *dieffenbachiae* Isolated from *Anthurium andraeanum* in China

**DOI:** 10.3390/pathogens7040085

**Published:** 2018-11-06

**Authors:** Shuang-Chun Li, Wei-Da Zeng, Xing-Wei Li, Xiao-Yun Zhou, Qiong-Guang Liu

**Affiliations:** 1 State Key Laboratory of Conservation and Utilization of Subtropical Agro-Bioresources, Guangdong Province Key Laboratory of Microbial Signals and Disease Control, College of Agriculture, South China Agricultural University, Guangzhou 510642, China; cherrylee92@foxmail.com (S.-C.L.); xingwli@163.com (X.-W.L.); 2 Guangzhou Flower Research Centre, Guangzhou 510360, China; weidaz@126.com

**Keywords:** *Anthurium andraeanum*, bacterial blight, *Xanthomonas axonopodis* pv. *dieffenbachiae*, pathotype, China

## Abstract

Anthurium blight, caused by *Xanthomonas axonopodis* pv. *dieffenbachiae* (*Xad*), is one of the most serious diseases of *Anthurium andraeanum.* However, little is known about variations in virulence between *Xad* pathotypes. Here, we examined the virulence of 68 *Xad* strains isolated from 30 anthurium plants from five regions of China against five different anthurium cultivars. Seven bacterial pathotypes were identified based on disease index and incidence analyses following foliar spray or leaf-clip inoculation. The resulting disease susceptibility patterns for pathotypes I–VII were RRRSS, RRSRS, RSRSR, RRSSS, RSSRS, RSSSS, and SSSSS, respectively. Overall, 72% of tested strains belonged to pathotypes VI or VII and were highly virulent. A further 22.1% of strains showed medium-level virulence and were classed as pathotype III, IV, or V, while the remaining 5.9% of strains were pathotype I or II, showing low virulence. Further analysis revealed differences in the virulence of *Xad* strains from the same anthurium cultivar, with variation also observed in pathovars associated with the same cultivar from different areas. Our results reveal the diversity and complexity of the *Xad* population structure in China and suggest that investigation of *Xad* pathotypes provides useful information to guide the identification and use of resistant varieties of *A. andraeanum*.

## 1. Introduction

*Anthurium andraeanum* is a tropical, perennial, evergreen plant that is highly sought after because of its colorful flowers and unique leaf shape. *A. andraeanum* is widely planted in many countries, including China, with commercial-scale growing operations found in Guangdong, Zhejiang, Yunnan, Fujian, and Hainan provinces. However, the production and development of *A. andraeanum* are seriously impacted by a bacterial blight disease caused by *Xanthomonas axonopodis* pv. *dieffenbachiae* (*Xad*). Anthurium blight was first reported in Brazil in 1960 and subsequently in Hawaii in 1971 [1]. The disease was responsible for the decline of the Hawaiian anthurium industry in the 1980s [2] and the Caribbean anthurium industry in the 1990s [3]. Due to its devastating effects, *Xad* is considered a quarantine pest by the European and Mediterranean Plant Protection Organization, the Caribbean Plant Protection Commission, and by several independent countries [4].

Anthurium blight is easily transmitted through excess water run-off, infected soil, and direct contact with infected plant materials and tools [5,6]. Many studies have focused on the identification, classification, molecular detection, and physiological and biochemical characterization of *Xad*, as well as on potential disease control measures [7,8,9,10]. Physical measures to control the disease have been suggested [11] but they are expensive and require capital intensive infrastructure and good management practices, such as daily removal of infected leaves, cut flowers, and plants [12]. Several chemicals have been screened for their potential to control the disease [13], however, widespread application of pesticides leads to environmental pollution and drug resistance, amongst other issues.

It is generally agreed that the most cost-effective approach to controlling anthurium blight is the use of resistant cultivars [12]. A previous study suggested that Julia and Gemini were the most resistant cultivars, while Hearts Desire was the most susceptible to *Xad* infection among 15 tested anthurium cultivars [14]. In another study, eight anthurium cultivars, including Alii, ARCS, Kalapana, Marian Seefurth, Nitta, Pink Elf, Tropic Mist, and UH1060, were inoculated with *Xad* strain V108LRUH1. The results indicated that in susceptible cultivars, pathogenic bacteria spread rapidly into the front of the petiole, while bacteria affecting the resistant cultivars spread more slowly and rarely migrated into the petiole [15]. Screening of 10 anthurium cultivars naturally infected with bacteria in the field revealed that cultivars White Queen, Florida Red Ruffles, Florida Sweetheart, Candidum Jr., and Mrs. Arno Nehrling were resistant to bacterial blight in both greenhouse and field-based evaluations, making them good candidates for use in future breeding efforts to produce resistant cultivars [16]. The inheritance of foliar resistance to blight is quantitative, with a major role for additive genetic effects [17]. Differences in the virulence of *Xad* strains are directly linked to the resistance of different anthurium plants and little research has been conducted on *Xad* pathotypes [18]. We previously classified 66 *Xad* strains from Guangdong Province, China, into 14 genetic groups using repetitive element palindromic-polymerase chain reaction (Rep-PCR) analysis and found that *Xad* strains in China show abundant genetic variation. However, differences in virulence among the strains are less clear. In the present study, we determined the pathotypes of 68 *Xad* strains isolated from anthurium plants from various regions of China, identifying seven different pathotypes. Among these, pathotypes VI and VII were the most virulent and were also the predominant pathotypes of *Xad* strains in China. The results of the current study provide a basis for breeding resistant varieties of *A. andraeanum*.

## 2. Materials and Methods

Bacterial strains and origin: 68 *Xad* strains were isolated from infected *A. andraeanum*, which were collected from Guangzhou, Conghua, Shaoguan, Shunde, Xinfeng and Shanghai. Details of the *Xad* strains examined in this study are provided in Table 1.

Differential varieties of *A. andraeanum:* Five varieties of *A. andraeanum*, Vita, Red Victory, Pink Champion, Alabama and *A. andraeanum* ‘Arebo’, all of which have shown different levels of resistance to *Xad* during the planting process for many years, were selected as differential hosts. The anthurium seedlings were planted in pots and were inoculated at the fourth-leaf stage.

Inoculation of *A. andraeanum* seedlings: Suspensions of *Xad* were inoculated onto anthurium seedlings by both leaf-cutting and foliar spray application. For leaf-cutting inoculation, the tips of two similarly-sized leaves from each plant were cut off using sterilized scissors and the bacterial suspensions were then evenly sprayed onto the wounded leaves. For spraying inoculation, the bacterial suspensions were sprayed directly onto unwounded leaves. Control plants were inoculated with sterile water. Each of the 68 *Xad* strains was individually inoculated onto the leaves of the five different varieties of *A. andraeanum*, with each bacterial strain inoculated onto 10 seedlings of each *A. andraeanum* variety. All bacterial suspensions were adjusted to a concentration of 1 × 10^8^ colony-forming units/mL. The inoculated anthurium seedlings were maintained in a greenhouse at 28 °C and >90% relative humidity.

Evaluation of disease severity: Disease progression was monitored weekly over the course of the experiment, with incidence and disease index values analyzed at 30 days post-inoculation.

The incidence of disease was equal to the number of diseased leaves divided by the number of total leaves ×100%.

The grading standard of disease severity was as follows:

- Level 0: No diseased spots on the leaf.
- Level 1: The length of diseased spots was 2–3 cm or the area of the diseased spot was less than 10% of the leaf.
- Level 3: The length of diseased spots was less than 1/4 of the leaf or the area of the diseased spot was less than 20% of the leaf.
- Level 5: The length of diseased spots was 1/4 and less than 1/2 of the leaf or the area of the diseased spot was between 20–49% of the leaf.
- Level 7: The length of diseased spots was 1/2 and less than 3/4 of the leaf or the area of the diseased spot was between 50–74% of the leaf.
- Level 9: The length of diseased spots was 3/4 or more of the leaf or the area of the diseased spot was over 75% of the leaf.

The disease index (DI), representing both the disease incidence and symptom severity, was calculated using the formula: DI = (∑Di × Dd)/(Mi × Md) × 100, where Di is number of diseased leaves in a disease grade, Dd is the rating scale of the corresponding disease grade, Mi is the total number of leaves observed, and Md is the rating scale of the maximum disease grade.

Data integration and normalization: To more accurately classify the results of the experimental data, data normalization was conducted. Based on the experimental design and the obtained results, there were four groups of data: Disease incidence and disease index from both the foliar spray inoculation and the leaf-cutting inoculation. The data from each of the four groups were normalized and weighted for parametric analysis and an analytical hierarchy process was adopted.

According to the order of the original data, in most cases, the rank statistics of the incidence and disease indexes of the inoculated plants could be fitted using a discrete exponential analysis model as well as a logarithmic normalization method. Using A to represent the incidence coefficient, B for the disease index coefficient, a for the incidence rate, and b for the disease index, the following formulas were used:

A = log_100_(a × 100 + 1), B = log_100_(b + 1), (A∈[0:1] B∈[0:1]).

**Figure 1:**
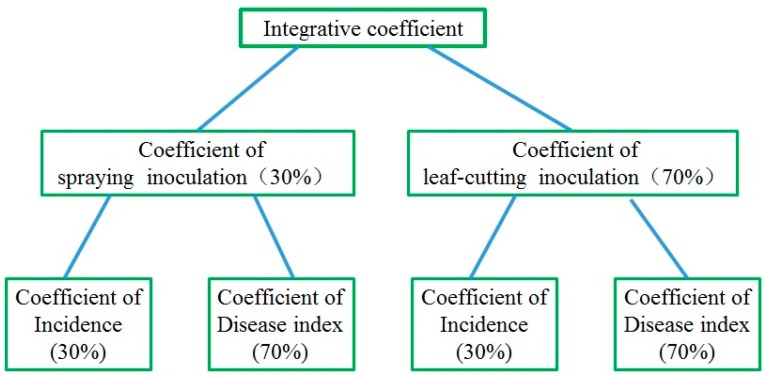
The hierarchy frame and weighting (%) of the integrative coefficient. Coefficients at each level: Spraying/leaf-cutting inoculation coefficient = 0.3 × incidence coefficient + 0.7 × disease index coefficient. Integrated coefficient = 0.3 × coefficient of spraying inoculation + 0.7 × coefficient of leaf-cutting inoculation.

Hierarchical weightings: Using a simplified analytical hierarchy process, the weighting of each group of data at each level was determined. Usually, the determination of virulence and physiological race of plant pathogenic bacteria is based on wound inoculation, with the disease index being an important standard. Therefore, when integrating the results of wound and spray inoculation, we gave wound inoculation a higher weighting (70%) than spray inoculation (30%), with the disease index given a weighting of 70% and the incidence a weighting of 30% (Figure 1).

Data classification and resistance/susceptibility determination: The integrated coefficient values were calculated according to the above formula, which then allowed each pathotype to be classified as either resistant (R) or susceptible (S). A pathotype was classified as resistant when the integrated coefficient value was between 0 and 0.5, while susceptible pathotypes were those with an integrated coefficient between 0.5 and 1. In this way, the corresponding pathotype of each *Xad* strain was obtained via statistical analysis and subsequently classified.

## 3. Results

### 3.1. Effects of Inoculation Method on the Virulence of Xanthomonas axonopodis pv. dieffenbachiae

At 30 days post-inoculation, differences in disease incidence and disease index were noted among the different anthurium varieties and *Xad* strains. The incidence of disease in the five anthurium varieties following foliar spray inoculation ranged from 0–38.89%, with disease index values ranging from 0–31.48. In contrast, following leaf-cut inoculation, the disease incidence ranged from 6.25–100%, with disease index values ranging from 0.69–98.52. The lowest average disease incidence and index values (4.11% and 0.85, respectively) were calculated for resistant the *A. andraeanum* variety Vita, while the highest average disease incidence and index values (78.2% and 57.42, respectively) were determined for the susceptible variety, *A. andraeanum* ‘Arebo’. However, in the leaf-cutting inoculation experiment, *Xad* strains showed differences in virulence against different anthurium varieties, while the same variety of *A. andraeanum* could also show different levels of resistance to different *Xad* strains. Overall, the disease incidence and index values were generally higher in the leaf-cutting assay compared with those obtained following foliar spray inoculation (Table 2).

### 3.2. Determination of the Integrated Coefficient Values and Pathotypes of Xad Strains

To allow for combined analysis of the leaf-cutting and foliar spray-based inoculation data, each of the data sets was given separate hierarchical weightings. The leaf-cutting inoculation data was given a rating of 70%, while the spraying inoculation data was given a weighting of 30%. In addition to considering the disease index, the incidence of disease was also considered in our analyses. As such, the disease index was assigned a weighting of 70%, while the disease incidence was given a weighting of 30%. Based on these weightings, the final integrated coefficient was calculated. As the normalized logarithmic values of the coefficients were used, the data showed a linear distribution with values between 0 and 1 (Table S1). In this study, the integrated coefficient values were used to classify pathotypes as either disease resistant (R) or susceptible (S), with values between 0 and 0.5 classified as resistant and those between 0.5 and 1 indicating a susceptible pathotype. After the determination of the resistant/susceptible phenotypes, the corresponding pathogenic type of each strain could be obtained through statistical analysis and computer-based classification. Based on these analyses, we determined that the 68 *Xad* strains could be divided into seven pathotypes. The phenotypic characteristics of pathotype I, II, III, IV, V, VI and VII on the five anthurium differentiation varieties (Vita, Red Victory, Pink Champion, Alabama, *A. andraeanum* ‘Arebo’) were: RRRSS, RRSRS, RRSSS, RSRSR, RSSRS, RSSSS and SSSSS, respectively (Table 3).

### 3.3. Pathotype Analysis

The results indicated that *Xad* strains belonging to pathotype VI or VII were the most prevalent and also the most virulent. Overall, 72% of the tested strains belonged to pathotype VI or VII. Of these, 29 strains isolated from 21 anthurium cultivars from five regions of China were classified as pathotype VI, while 20 strains from 15 anthurium cultivars from six regions were classified as pathotype VII. Pathotype I and II strains were less prevalent and showed the lowest levels of virulence in this study. These two pathotypes accounted for only 5.9% of all strains, amongst which, only *Xad 46* strain, isolated from the *A. andraeanum* cultivar Sandy in the Conghua region, was classified as pathotype I. Pathotype III, IV, and V strains were moderately virulent and accounted for 22.1% of the tested strains. Overall, eight, one, and six *Xad* strains were classified as pathotype III, IV, and V, respectively. Therefore, we hypothesize that the majority of *Xad* strains in China display a high level of virulence.

Interestingly, variation was observed in the virulence of *Xad* strains isolated from the same anthurium cultivar. For example, seven *Xad* strains isolated from the *A. andraeanum* cultivar Alabama were divided amongst four pathotypes (II, III, VI and VII), while nine strains isolated from Dakota cultivar plants were spread amongst pathotypes III, VI, and VII. In addition, we noted differences in the types and virulence levels of strains isolated from the same cultivar but from different geographical origins (Table 4). However, there was variation in strains from the same geographical origin. For example, in the Conghua area, 53 *Xad* strains isolated from 26 *A. andraeanum* cultivars were divided amongst the seven pathotypes (I~VII) (Table 5) but pathotype VI and VII strains were predominant.

## 4. Discussion

Understanding the pathogenicity and differences in virulence of plant-pathogenic bacteria is a key factor in epidemic forecasting and in breeding disease-resistant plant varieties. Different anthurium cultivars display varying susceptibilities to the foliar and systemic phases of bacterial blight infection [15]. Resistance to both phases of infection is required to reduce the damaging effects of the pathogen. Understanding the genetic basis of both the resistance to blight in anthurium and the virulence of *Xad* is important for selecting parents for breeding and for designing an efficient anthurium breeding program [19]. A comprehensive analysis of 175 *Xanthomonas axonopodis* pv. *dieffenbachiae* strains isolated from 10 different Araceae hosts was previously conducted to assess pathogen variation [20]. The isolates were subjected to repetitive extragenic palindromic sequence polymerase chain reaction (Rep-PCR) analysis and four major phylogenetic clusters were generated. The results indicated that isolates grouped into cluster I, which were isolated primarily from anthurium cultivars, likely constituted an undescribed pathovar [20]. We later classified 66 *Xad* strains isolated from 27 anthurium cultivars from Guangdong Province, China, into 14 genetic groups based on Rep-PCR analysis and identified obvious genetic differentiation amongst *Xad* strains in China [18]. We further investigated the virulence of 68 *Xad* strains isolated from 30 different anthurium cultivars from China in the current study via foliar-spray and leaf-cutting inoculation of five different anthurium varieties. Our results indicated that the 68 *Xad* strains belonged to seven different pathotypes, showing some correlation with genetic groups previously identified by Rep-PCR analysis [18]. Strains belonging to pathotype VI, isolated from 19 anthurium cultivars, and pathotype VII, isolated from 14 cultivars, were the most prevalent, accounting for 42.6% and 29.4% of all strains, respectively. Importantly, these two pathotypes were also associated with the highest levels of virulence in the current study.

We noted differences in the virulence of *Xad* strains isolated from the same cultivar but from different geographical locations (Table 4). Seven strains from the *A. andraeanum* cultivar Alabama belonged to four different pathotypes (II, III, VI, and VII), while nine strains from the cultivar Dakota were divided amongst pathotypes III, VI, and VII. The results also suggested that the majority of *Xad* strains isolated from cultivars Alabama and Dakota were highly virulent. While the 53 strains isolated from the Conghua area of Guangdong Province showed a high degree of pathotype diversity (pathotypes I–VII), strains belonging to pathotypes VI and VII were the most prevalent. Therefore, to prevent an outbreak of anthurium blight in the Conghua area, anthurium cultivar breeding should be aimed at developing resistance to pathotype VI and VII *Xad* strains.

The inoculation method and selected disease severity scoring system are very important for assessing the virulence of pathogenic bacteria. Foliar infection occurs following pathogen entry into the leaves via hydathodes [21] or wounds [5]. In this study, two inoculation methods (leaf-cutting and spraying) were examined. The results showed that leaf-cutting was more effective than spraying for initiating disease, with some *Xad* strains showing virulence following leaf-cut inoculation but causing no symptoms in the foliar spray assays (Table 2). To allow for an integrated assessment of our results, we applied a weighting of 70% to the leaf-cut data versus 30% for the foliar spray data. Additionally, the disease index was given a weighting of 70%, while the incidence of disease was assigned a weighting of 30%. This allowed a final integrated assessment of coefficients and assignment of pathotypes into two categories: resistant and susceptible. Due to the integration of four sets of disease incidence and disease index data associated with two inoculation methods on five different varieties of *A. andraeanum*, we are confident that our results objectively and accurately reflect the virulence profiles of the 68 *Xad* strains.

## Figures and Tables

**Table 1:** *Xad* strains, cultivars and origin.

*Xad* Strains	*Anthurium cultivars*	Origin	*Xad* Strains	*Anthurium cultivars*	Origin
*Xad1*, *Xad2*	*A. feudleri*	Conghua	*Xad36*, *Xad37*	*A. andraeanum* ‘Ricado’	Conghua
*Xad3*~*Xad6*	*A. andraeanum* ‘Dakota’	Conghua	*Xad38*, *Xad39*	*A. andraeanum* ‘Kaxino’	Conghua
*Xad7*, *Xad8*	*A. itanhaense*	Conghua	*Xad40*, *Xad41*	*A. andraeanum* ‘Luosa’	Conghua
*Xad9~Xad13*	*A. andraeanum* ‘Sharade’	Conghua	*Xad42*	*A. andraeanum* ‘Windward’	Conghua
*Xad14*, *Xad15*	*A. andraeanum* ‘Senator’	Conghua	*Xad43*, *Xad44*	*A. andraeanum* ‘Baron’	Conghua
*Xad16*, *Xad17*	*A. andraeanum* ‘Madural’	Conghua	*Xad45*, *Xad46*	*A. andraeanum* ‘Sandy’	Conghua
*Xad18*, *Xad19*	*A. andraeanum* ‘Red Queen’	Conghua	*Xad47*, *Xad48*	*A. andraeanum* ‘Pink Champion’	Conghua
*Xad20*	*A. andraeanum* ‘Sun fire’	Conghua	*Xad49*, *Xad50*	*A. andraeanum* ‘Fantasy Love’	Conghua
*Xad21*, *Xad22*	*A. crassinervium*	Conghua	*Xad51*, *Xad52*	*A. andraeanum* ‘Alabama’	Conghua
*Xad23*, *Xad24*	*A. andraeanum* ‘Dovetail Red’	Conghua	*Xad53*	*A. andraeanum* ‘Mating’	Conghua
*Xad25*, *Xad26*	*A. andraeanum* ‘Catherine’	Conghua	*Xad54~Xad56*	*A. andraeanum* ‘Vito’	Sunde
*Xad27*	*A. andraeanum* ‘Pistache’	Conghua	*Xad57*	*A. andraeanum* ‘Alabama’	Sanshui
*Xad28*, *Xad29*	A040	Conghua	*Xad58*, *Xad59*	*A. andraeanum* ‘Dakota’	Shaoguan
*Xad30*, *Xad31*	*A. andraeanum* ‘Toscane’	Conghua	*Xad60~Xad62*	*A. andraeanum* ‘Alabama’	Xinfeng
*Xad32*, *Xad33*	*A. andraeanum* ‘Crystal Candle’	Conghua	*Xad63*, *Xad64*	*A. andraeanum* ‘Ardour’	Guangzhou
*Xad34*, *Xad35*	*A. andraeanum* ‘Arebo’	Conghua	*Xad65*	*A. andraeanum* ‘Alabama’	Guangzhou
			*Xad66~Xad68*	Dakota	Shanghai

**Table 2:** Disease investigation after spraying and leaf-cutting inoculation after 30 days.

*Xad* Strains	Varieties (Disease Index/Disease Incidence%)				
	*A. andraeanum* ‘Vita’	*A. andraeanum* ‘Red Victory’	*A. andraeanum* ‘Pink Champion’	*A. andraeanum* ‘Alabama’	*A. andraeanum* ‘Arebo’
*Xad1*	0.00/0.00 ^a^	0.65/5.88	0.74/6.67	0.46/4.17	14.81/14.81
	13.58/44.44 ^b^	33.33/88.89	67.28/100.00	3.70/33.33	34.64/100.00
*Xad2*	3.09/5.59	0.00/0.00	0.51/4.55	15.79/15.79	0.74/6.67
	24.31/43.75	45.83/87.50	47.22/87.50	37.78/60.00	26.39/75.00
*Xad3*	0.00/0.00	3.70/22.22	4.89/12.00	0.51/4.55	2.02/9.09
	11.11/46.67	25.93/86.67	75.69/93.75	28.57/42.86	25.69/68.75
*Xad4*	1.39/6.25	0.00/0.00	0.53/4.76	1.43/6.45	1.35/12.12
	0.69/6.25	8.15/46.67	42.86/100.00	14.81/53.33	18.51/73.33
*Xad5*	1.11/10.00	6.88/33.33	26.39/37.50	31.48/38.89	9.66/34.78
	31.11/53.33	48.15/60.00	59.26/80.00	50.37/66.67	68.89/86.67
*Xad6*	0.00/0.00	0.00/0.00	0.00/0.00	0.53/4.76	21.53/43.75
	20.74/26.67	30.37/46.67	46.67/60.00	16.67/25.00	76.39/100.00
*Xad7*	1.15/3.44	3.85/3.85	0.40/3.57	0.43/3.85	3.23/16.13
	9.63/46.67	20.00/73.33	69.84/85.71	57.78/80.00	43.58/43.58
*Xad8*	0.00/0.00	5.56/27.78	9.44/15.00	6.35/9.52	4.70/42.31
	1.48/13.33	57.04/100.00	49.31/68.75	25.40/42.86	39.26/86.67
*Xad9*	0.00/0.00	1.31/11.76	0.00/0.00	0.00/0.00	1.35/12.12
	2.96/26.67	6.67/46.67	42.96/53.33	25.69/56.25	18.75/68.75
*Xad10*	3.24/12.50	4.83/26.09	0.00/0.00	1.93/8.70	9.47/25.93
	17.78/40.00	39.32/92.31	64.10/84.62	56.41/92.31	65.19/100.00
*Xad11*	0.00/0.00	0.00/0.00	0.00/0.00	0.00/0.00	2.56/23.08
	5.93/40.00	50.00/100.00	20.00/33.33	23.46/55.56	4.27/38.46
*Xad12*	1.65/7.41	1.78/8.00	2.81 11.11	0.41/3.70	6.30/16.67
	5.56/50.00	33.33/100.0	76.19/100.0	16.24/38.46	30.37/86.70
*Xad13*	0.00/0.00	4.94/33.33	4.00/12.00	2.78/5.00	6.76/17.39
	8.33/50.00	35.56/66.67	69.44/87.50	22.96/73.33	22.22/75.00
*Xad14*	1.15/3.45	1.23/11.11	14.29/14.29	19.05/19/05	12.12/12.12
	15.28/50.00	54.17/100.00	90.12/100.00	54.81/93.33	40.17/100.00
*Xad15*	0.00/0.00	1.11/10.00	2.22/20.00	0.53/4.76	2.22/20.00
	2.61/22.22	18.52/73.33	63.89/100.00	11.11/37.50	24.79/81.25
*Xad16*	0.00/0.00	1.39/12.50	0.00/0.00	0.37/3.33	0.72/6.45
	2.96/26.67	22.96/53.33	57.78/66.67	35.56/66.67	27.41/73.33
*Xad17*	1.71/7.69	0.43/3.85	0.00/0.00	0.85/7.69	4.37/25.00
	8.33/37.50	29.63/80.00	86.51/92.86	49.63/73.33	48.89/66.67
*Xad18*	0.43/3.84	0.00/0.00	0.85/7.69	0.00/0.00	4.76/21.43
	6.67/33.33	15.56/60.00	70.94/84.62	23.70/66.67	25.49/76.47
*Xad19*	0.62/5.56	1.75/15.79	0.00/0.00	1.39/12.50	15.46/43.48
	17.78/53.33	20.00/60.00	33.33/73.33	27.41/33.33	42.86/85.71
*Xad20*	1.85/8.33	0.82/7.41	1.01/9.09	13.19/18.75	0.93/8.33
	2.22/20.00	9.52/57.14	58.33/75.00	37.91/58.82	35.42/68.75
*Xad21*	1.59/4.76	5.88/5.88	0.00/0.00	2.88/18.52	1.23/11.11
	1.48/13.33	6.67/33.33	51.11/60.00	14.07/46.67	8.89/40.00
*Xad22*	0.44/4.00	0.97/8.70	0.00/0.00	0.89/8.00	3.07/20.69
	0.74/6.67	12.59/73.33	55.56/73.33	16.67/50.00	19.26/80.00
*Xad23*	0.00/0.00	2.02/9.09	0.62/5.56	0.00/0.00	1.67/15.00
	3.47/18.75	3.92/25.53	61.48/73.33	9.63/20.00	28.47/93.75
*Xad24*	2.65/14.29	3.97/14.29	1.28/3.85	12.35/22.22	15.95/23.33
	8.15/33.33	11.11/46.57	51.85/80.00	14.29/28.57	29.86/81.25
*Xad25*	0.97/8.70	4.63/8.88	0.00/0.00	1.33/12.00	0.00/0.00
	16.67/38.89	51.11/100.00	65.81/84.62	35.56/81.25	31.94/87.50
*Xad26*	0.41/3.70	0.43/3.85	0.00/0.00	4.98/17.24	3.37/24.24
	14.81/40.00	26.50/100.00	58.73/85.71	25.40/78.57	35.56/93.33
*Xad27*	0.00/0.00	0.00/0.00	5.22/11.76	0.00/0.00	1.75/15.79
	7.64/35.00	20.00/76.47	82.22/100.00	11.81/17.65	43.06/64.29
*Xad28*	0.00/0.00	0.00/0.00	0.00/0.00	0.00/0.00	0.00/0.00
	10.37/66.67	38.52/78.57	51.11/73.33	51.28/92.31	20.74/66.67
*Xad29*	0.00/0.00	0.82/7.41	4.55/4.55	0.00/0.00	0.85/7.69
	1.48/13.33	18.52/57.14	45.24/60.00	1.48/13.33	30.37/73.33
*Xad30*	0.00/0.00	1.31/11.76	1.11/10.00	28.57/11.76	1.85/16.67
	3.89/25.00	15.56/66.67	75.00/100.00	51.63/76.47	34.72/100.00
*Xad31*	1.33/4.00	1.33/12.00	0.00/0.00	0.41/3.70	4.37/17.86
	8.89/40.00	29.63/80.00	90.64/100.00	42.22/73.33	37.50/87.50
*Xad32*	0.00/0.00	0.00/0.00	0.00/0.00	0.48/4.35	1.48/13.33
	13.89/37.50	34.26/58.33	29.17/50.00	33.33/50.00	16.30/53.33
*Xad33*	3.51/10.53	0.51/4.55	5.31/13.04	1.85/16.67	3.03/27.27
	3.47/18.75	19.05/85.71	65.87/86.67	19.26/53.33	30.37/100.00
*Xad34*	0.00/0.00	0.46/4.17	5.80/17.49	2.53/13.64	0.00/0.00
	3.70/20.00	42.22/73.33	98.41/100.00	26.39/62.50	33.33/100.00
*Xad35*	0.53/4.76	0.46/4.17	2.65/14.29	1.33/12.00	3.29/22.22
	4.44/40.00	28.89/86.67	57.04/86.67	26.67/80.00	20.00/86.67
*Xad36*	2.22/13.33	1.01/9.09	4.70/19.23	0.00/0.00	0.00/0.00
	5.19/33.33	14.81/66.67	53.33/80.00	13.33/40.00	21.48/73.33
*Xad37*	0.00/0.00	0.00/0.00	0.00/0.00	10.00/10.00	2.31/4.17
	3.92/35.29	62.50/87.50	94.44/100.00	46.53/68.74	37.04/100.00
*Xad38*	3.33/30.00	1.59/14.29	0.00/0.00	23.08/23.08	0.00/0.00
	9.72/50.00	12.59/56.67	24.44/33.33	35.56/48.00	42.96/80.00
*Xad39*	0.41/3.70	0.44/4.00	1.23/3.70	4.94/14.81	3.81/22.86
	3.70/33.33	21.48/73.33	72.22/83.33	36.51/85.71	48.72/100.00
*Xad40*	0.00/0.00	0.53/4.76	1.45/4.35	12.82/23.08	4.55/13.64
	5.19/33.33	25.40/71.43	70.37/83.33	13.49/35.71	37.50/87.50
*Xad41*	2.22/10.00	2.42/13.04	1.59/7.69	0.85/7.69	4.53/25.93
	6.94/37.50	19.66/84.62	51.85/66.67	18.25/50.00	35.04/84.62
*Xad42*	0.00/0.00	0.00/0.00	0.00/0.00	0.46/4.17	1.48/13.33
	3.70/20.00	14.07/73.33	43.65/64.29	10.42/43.75	14.07/60.00
*Xad43*	0.43/3.85	0.00/0.00	0.79/7.14	0.00/0.00	0.71/6.90
	7.41/40.00	6.67/33.33	74.81/100.00	27.08/56.25	25.93/73.33
*Xad44*	1.52/13.64	2.22/10.00	3.89/5.00	0.00/0.00	0.93/8.33
	5.56/37.50	12.50/62.50	82.22/100.00	31.85/73.33	30.07/94.12
*Xad45*	0.37/3.33	0.00/0.00	1.11/10.00	0.43/3.85	0.74/6.67
	5.19/33.33	28.15/80.00	60.00/86.67	29.91/53.85	22.22/80.00
*Xad46*	0.00/0.00	0.00/0.00	0.00/0.00	2.56/7.69	2.22/12.00
	3.70/33.33	8.89/53.33	11.11/33.33	11.11/28.57	15.56/60.00
*Xad47*	0.69/6.25	1.23/11.11	0.48/4.35	1.11/10.00	0.48/4.35
	7.41/44.44	29.63/80.00	81.70/100.00	35.29/70.59	37.04/100.00
*Xad48*	0.82/7.41	0.48/4.35	0.00/0.00	1.28/11.54	2.67/16.00
	2.78/25.00	24.79/100.00	62.75/82.36	69.84/100.00	33.33/75.00
*Xad49*	1.45/4.35	1.10/9.09	0.00/0.00	1.06/8.70	3.54/31.82
	5.23/25.53	17.78/66.67	60.00/73.33	35.42/56.25	29.63/80.00
*Xad50*	2.32/11.11	0.00/0.00	2.22/4.17	0.40/3.57	1.11/10.00
	21.48/46.67	25.40/85.71	93.06/100.00	21.48/46.67	63.70/93.33
*Xad51*	0.00/0.00	5.31/13.04	0.00/0.00	25.69/31.25	8.99/23.81
	55.56/100.00	83.70/100.00	88.19/93.75	28.15/66.67	67.44/100.00
*Xad52*	0.00/0.00	0.00/0.00	0.00/0.00	0.53/4.76	0.00/0.00
	0.69/6.25	30.56/75.00	71.85/100.00	40.52/70.59	45.68/100.00
*Xad53*	0.00/0.00	0.00/0.00	1.67/5.00	1.52/13.64	2.34/15.79
	2.22/20.00	2.96/26.67	38.46/69.23	20.74/53.33	9.63/33.33
*Xad54*	0.37/3.33	0.00/0.00	0.85/7.69	0.00/0.00	1.53/13.79
	3.92/35.29	5.56/35.71	15.56/33.33	31.25/56.25	24.79/69.23
*Xad55*	0.00/0.00	4.86/18.75	4.83/8.70	4.27/23.08	4.89/28.00
	9.03/56.25	38.16/100.00	82.22/100.00	27.41/73.33	24.44/86.67
*Xad56*	2.47/7.41	0.00/0.00	0.00/0.00	3.54/4.55	1.48/13.33
	9.52/28.57	34.81/100.00	90.28/100.00	22.96/46.67	33.33/87.50
*Xad57*	0.82/7.41	0.51/4.55	0.00/0.00	0.00/0.00	2.53/22.73
	1.31/11.76	7.64/68.75	40.12/61.11	15.69/58.82	13.58/66.67
*Xad58*	0.62/5.56	0.62/5.56	1.48/13.33	0.00/0.00	7.11/24.00
	3.92/23.53	9.63/60.00	79.26/100.0	39.87/76.47	19.26/80.00
*Xad59*	0.00/0.00	0.48/4.34	0.41/3.70	1.17/10.53	1.65/14.81
	21.57/88.24	15.56/73.33	41.67/50.00	51.39/75.00	61.40/100.00
*Xad60*	0.00/0.00	0.93/8.33	1.45/13.04	0.00/0.00	3.57/32.14
	4.86/43.75	10.37/53.33	45.93/66.67	40.28/50.00	11.11/64.71
*Xad61*	0.00/0.00	0.00/0.00	0.62/5.56	0.51/4.55	2.22/20.00
	10.42/43.75	11.85/53.33	32.40/66.67	23.61/50.00	9.72/50.00
*Xad62*	9.52/9.52	0.00/0.00	0.00/0.00	0.00/0.00	1.15/10.34
	23.61/75.0	40.74/86.67	45.83/75.00	51.39/75.00	39.58/81.25
*Xad63*	0.00/0.00	0.00/0.00	1.11/10.00	0.00/0.00	0.48/4.35
	2.22/20.00	26.39/75.00	35.56/53.33	4.44/26.67	20.83/75.00
*Xad64*	0.93/8.33	0.00/0.00	0.00/0.00	0.00/0.00	0.82/7.41
	2.97/26.67	17.04/60.00	67.41/86.67	32.48/61.54	24.31/68.75
*Xad65*	2.47/11.11	4.94/22.22	10.37/13.33	1.75/15.79	1.33/12.00
	17.78/53.33	57.14/85.71	95.07/100.00	28.89/60.00	46.83/92.86
*Xad66*	0.00/0.00	0.85/7.69	8.00/8.00	5.98/15.38	2.42/21.74
	14.07/60.00	37.50/87.50	98.52/100.00	40.00/66.67	65.93/100.00
*Xad67*	0.93/8.33	0.51/4.55	0.69/6.25	1.11/10.00	2.22/20.00
	1.96/17.65	8.15/60.00	35.71/64.29	38.56/76.47	23.53/82.35
*Xad68*	0.43/3.85	2.88/11.11	0.47/4.17	1.33/4.00	10.32/39.29
	15.97/56.25	25.64/92.31	64.29/92.86	27.78/83.33	55.56/87.50

^a^ Disease index/disease incidence % by spraying inoculation. ^b^ Disease index/disease incidence % by leaf-cutting inoculation.

**Table 3:** The pathotypes of 68 *Xad* strains.

Pathotypes	Differentiation Varieties					*Xad* Strains	Total Strains
	*A. andraeanum* ‘Vita’	*A. andraeanum* ‘Red Victory’	*A. andraeanum* ‘Pink Champion’	*A. andraeanum* ‘Alabama’	*A. andraeanum* ‘Arebo’		
I	R ^1^	R	R	S ^2^	S	*Xad46*	1
II	R	R	S	R	S	*Xad23*, *Xad57*, *Xad42*	3
III	R	R	S	S	S	*Xad4*, *Xad18*, *Xad43*, *Xad53*, *Xad54*, *Xad61*, *Xad64*, *Xad67*	8
IV	R	S	R	S	R	*Xad11*	1
V	R	S	S	R	S	*Xad1*, *Xad15*, *Xad27*, *Xad29*, *Xad36*, *Xad63*	6
VI	R	S	S	S	S	*Xad3*, *Xad6*, *Xad8*, *Xad9*, *Xad12*, *Xad13*, *Xad16*, *Xad20*, *Xad21*, *Xad22*, *Xad28*, *Xad30*, *Xad32*, *Xad33*, *Xad34*, *Xad35*, *Xad37*, *Xad39*, *Xad40*, *Xad44*, *Xad45*, *Xad47*, *Xad48*, *Xad49*, *Xad52*, *Xad55*, *Xad58*, *Xad60*, *Xad66*	29
VII	S	S	S	S	S	*Xad2*, *Xad5*, *Xad7*, *Xad10*, *Xad14*, *Xad17*, *Xad19*, *Xad24*, *Xad25*, *Xad26*, *Xad31*, *Xad38*, *Xad41*, *Xad50*, *Xad51*, *Xad56*, *Xad59*, *Xad62*, *Xad65*, *Xad68*	20

^1^ R: resistant. ^2^ S: susceptible.

**Table 4:** Pathotypes of *Xad* strains from Alabama and Dakota in different geographical origin.

*Xad* Strains	*Anthurium cultivars*	Origin	Pathotypes
*Xad57*	*A. andraeanum* ‘Alabama’	Sanshui	II
*Xad61*	*A. andraeanum* ‘Alabama’	Xinfeng	III
*Xad60*	*A. andraeanum* ‘Alabama’	Xinfeng	VI
*Xad52*	*A. andraeanum* ‘Alabama’	Conghua	VI
*Xad62*	*A. andraeanum* ‘Alabama’	Xinfeng	VII
*Xad65*	*A. andraeanum* ‘Alabama’	Guangzhou	VII
*Xad51*	*A. andraeanum* ‘Alabama’	Conghua	VII
*Xad4*	*A. andraeanum* ‘Dakota’	Conghua	III
*Xad67*	*A. andraeanum* ‘Dakota’	Shanghai	III
*Xad3*, *Xad6*	*A. andraeanum* ‘Dakota’	Conghua	VI
*Xad66*	*A. andraeanum* ‘Dakota’	Shanghai	VI
*Xad58*	*A. andraeanum* ‘Dakota’	Shaoguan	VI
*Xad5*	*A. andraeanum* ‘Dakota’	Conghua	VII
*Xad68*	*A. andraeanum* ‘Dakota’	Shanghai	VII
*Xad59*	*A. andraeanum* ‘Dakota’	Shaoguan	VII

**Table 5:** Pathotypes of 53 *Xad* strains from 26 cultivars in Conghua area.

*Xad* Strains (*Anthurium cultivars*)	Pathotypes	Total Strains
*Xad46* (Sandy).	I	1
*Xad23* (Dovetail Red), *Xad42* (Windward).	II	2
*Xad4* (Dakota), *Xad18* (Red Queen), *Xad43* (Baron), *Xad53* (Mating).	III	4
*Xad11* (Sharada).	IV	1
*Xad1* (*A. ieudleri*), *Xad15* (Senator), *Xad27* (Pistache), *Xad29* (A040), *Xad36* (Ricado).	V	5
*Xad3* (Dakota), *Xad6* (Dakota), *Xad8* (*A. itanhaense*), *Xad9* (Sharada), *Xad12* (Sharada), *Xad13* (Sharada), *Xad16* (Madural), *Xad20* (Sun fire), *Xad21* (*A. cassinervium*), *Xad22* (*A. cassinervium*), *Xad28* (A040), *Xad30* (Toscane), *Xad32* (Crystal Candle), *Xad33* (Crystal Candle), *Xad34* (Arebo), *Xad35* (Arebo), *Xad37* (Ricado), *Xad39* (Kaxino), *Xad40* (Luosa), *Xad44* (Baron), *Xad45* (Sandy), *Xad47* (Pink Champion), *Xad48* (Pink Champion), *Xad49* (Fantasy Love), *Xad52* (*A. andraeanum* ‘Alabama’).	VI	25
*Xad2* (*A. ieudleri*), *Xad7* (*A. itanhaense*), *Xad10* (Sharada), *Xad14* (Senator), *Xad17* (Madural), *Xad19* (Red Queen), *Xad25* (Catherine), *Xad26* (Catherine), *Xad31* (Toscane), *Xad38* (Kaxino), *Xad41* (Luosa), *Xad24* (Dovetail Red), *Xad5* (Dakota), *Xad50* (Fantasy Love), *Xad51* (*A. andraeanum* ‘Alabama’).	VII	15

## Supplementary Materials

The following are available online at http://www.mdpi.com/2076-0817/7/4/85/s1, Table S1 showed the integrative coefficients and resistant/susceptible phenotypes of *Xad* strains on 5 anthurium varieties inoculation.
