# Supplementary material for: Pathotypes of Xanthomonas axonopodis pv. dieffenbachiae Isolated from Anthurium andraeanum in China
Source: Pathogens. 2018 Nov 6;7(4):85. doi: 10.3390/pathogens7040085 (PMC6313323; doi:10.3390/pathogens7040085)
Supplement: Supplementary file 1 [file pathogens-07-00085-s001.pdf]

Table S1. The integrative coefficients and resistant/susceptible phenotypes of *Xad* strains on 5 anthurium varieties inoculation.

No. of *Xad*	Differentiation Varieties				
	A. andraeanum 'Vita'	*A. andraeanum* 'Red Victory'	*A. andraeanum* 'Pink Champion'	*A. andraeanum* 'Alabama'	*A. andraeanum* 'Arebo'
Xad1	0.459152[a]/S[b]	0.657805/S	0.742104/S	0.388081/R	0.824505/S
Xad2	0.647806/S	0.613707/S	0.68273/S	0.815589/S	0.631943/S
Xad3	0.441586/R	0.723536/S	0.838354/S	0.598686/S	0.665375/S
Xad4	0.246398/R	0.411764/R	0.680357/S	0.577807/S	0.625916/S
Xad5	0.653881/S	0.811786/S	0.92498/S	0.911019/S	0.885166/S
Xad6	0.47904/R	0.542862/S	0.59863/S	0.521742/S	0.953355/S
Xad7	0.509825/S	0.654155/S	0.713494/S	0.693237/S	0.753393/S
Xad8	0.218047/R	0.837108/S	0.819323/S	0.698202/S	0.792914/S
Xad9	0.297846/R	0.504614/S	0.584728/S	0.534022/S	0.624317/S
Xad10	0.631338/S	0.786112/S	0.647248/S	0.757361/S	0.87797/S
Xad11	0.375322/R	0.628808/S	0.485189/R	0.524185/S	0.497522/R
Xad12	0.489489/R	0.701277/S	0.813748/S	0.528328/S	0.76009/S
Xad13	0.416916	0.767825	0.816224	0.658079	0.726222
Xad14	0.558312/S	0.745403/S	0.921553/S	0.889233/S	0.824056/S
Xad15	0.280005/R	0.615214/S	0.798148/S	0.49944/R	0.690606/S
Xad16	0.297846/R	0.635192/S	0.625653/S	0.629203/S	0.633424/S
Xad17	0.51629/S	0.623882/S	0.682915/S	0.703006/S	0.78814/S
Xad18	0.437357/R	0.486129/R	0.746832/S	0.533402/S	0.7277/S
Xad19	0.568409/S	0.640179/S	0.572712/S	0.632385/S	0.866722/S
Xad20	0.380213/R	0.522842/S	0.7295/S	0.808679/S	0.669324/S
Xad21	0.31769/R	0.541375/S	0.608105/S	0.618175/S	0.521397/S
Xad22	0.212373/R	0.56951/S	0.625837/S	0.575959/S	0.67926/S
Xad23	0.29536/R	0.437798/R	0.710603/S	0.390334/R	0.693369/S
Xad24	0.540577/S	0.604163/S	0.710323/S	0.677304/S	0.814324/S
Xad25	0.569063/S	0.790866/S	0.650007/S	0.696618/S	0.576271/S
Xad26	0.52086/S	0.622476/S	0.638664/S	0.725914/S	0.756958/S
Xad27	0.392858/R	0.522303/S	0.852837/S	0.404771/R	0.722125/S
Xad28	0.450853/R	0.590799/S	0.617118/S	0.627835/S	0.51982/S
Xad29	0.218047/R	0.588616/S	0.740548/S	0.218047/R	0.652072/S
Xad30	0.317453/R	0.602485/S	0.773828/S	0.886901/S	0.723084/S
Xad31	0.502998/S	0.677089/S	0.691165/S	0.654995/S	0.765155/S
Xad32	0.453833/R	0.565278/S	0.541791/S	0.61935/S	0.604211/S
Xad33	0.445301/R	0.588426/S	0.82626/S	0.634473/S	0.741552/S
Xad34	0.303497/R	0.659386/S	0.885885/S	0.682215/S	0.586694/S
Xad35	0.41716/R	0.627674/S	0.779896/S	0.666275/S	0.691514/S
Xad36	0.489818/R	0.583492/S	0.803065/S	0.452623/R	0.527662/S
Xad37	0.333309/R	0.646109/S	0.695488/S	0.807531/S	0.709616/S

Xad38	0.602712/S	0.576752/S	0.505597/S	0.82987/S	0.60294/SS
Xad39	0.383689/R	0.588197/S	0.74475/S	0.763391/S	0.797288/S
Xad40	0.35521/R	0.611181/S	0.750841/S	0.684422/S	0.761129/S
Xad41	0.515238/S	0.662917/S	0.723776/S	0.582934/S	0.766892/S
Xad42	0.303497/R	0.48511/R	0.594765/S	0.494633/R	0.594808/S
Xad43	0.455303/R	0.378021/R	0.756376/S	0.539424/S	0.628773/S
Xad44	0.486809/R	0.594532/S	0.820217/S	0.568023/S	0.666566/S
Xad45	0.409284/R	0.559227/S	0.74398/S	0.607073/S	0.617275/S
Xad46	0.32591/R	0.426002/R	0.426616/R	0.548615/S	0.618417/S
Xad47	0.479738/R	0.672729/S	0.744057/S	0.679482/S	0.661427/S
Xad48	0.377236/R	0.620074/S	0.643798/S	0.774185/S	0.720367/S
Xad49	0.435069/R	0.604476/S	0.633878/S	0.665214/S	0.739759/S
Xad50	0.639866/S	0.55179/S	0.804263/S	0.564084/S	0.753584/S
Xad51	0.639819/S	0.857864/S	0.685368/S	0.833715/S	0.876716/S
Xad52	0.146168/R	0.564774/S	0.666749/S	0.658829/S	0.619391/S
Xad53	0.263258/R	0.297846/R	0.687411/S	0.630002/S	0.553355/S
Xad54	0.387383/R	0.364444/R	0.54887/S	0.554156/S	0.660369/S
Xad55	0.429885/R	0.779403/S	0.842364/S	0.729536/S	0.736514/S
Xad56	0.531299/S	0.591185/S	0.690746/S	0.64712/S	0.69938/S
Xad57	0.292378/R	0.488929/R	0.583725/S	0.486071/R	0.62953/S
Xad58	0.389624/R	0.513134/S	0.795766/S	0.593153/S	0.724618/S
Xad59	0.536424/S	0.558912/S	0.636454/S	0.724104/S	0.775362/S
Xad60	0.361468/R	0.534064/S	0.719179/S	0.575151/S	0.632115/S
Xad61	0.432461/R	0.45386/R	0.639683/S	0.586028/S	0.575403/S
Xad62	0.73763/S	0.601035/S	0.606764/S	0.618701/S	0.699569/S
Xad63	0.263258/R	0.549695/S	0.66769/S	0.331633/R	0.589368/S
Xad64	0.391339/R	0.495238/R	0.653604/S	0.562169/S	0.624557/S
Xad65	0.629385/S	0.819177/S	0.907603/S	0.677739/S	0.731229/S
Xad66	0.476097/R	0.681821/S	0.886047/S	0.772218/S	0.807006/S
Xad67	0.342112/R	0.488918/R	0.6530 63/S	0.692261/S	0.685882/S
Xad68	0.545224/S	0.698048/S	0.714335/S	0.649538/S	0.869563/S

Note: a is integrative coefficients, b is resistant/susceptible phenotypes, R: resistant, S: susceptible.
